# An innovative method for surmounting the constrained torsional problems of stocky beams with arbitrary noncircular cross-sectional shapes and with arbitrary elastic material properties

**DOI:** 10.1016/j.mex.2021.101252

**Published:** 2021-01-26

**Authors:** Yaoqing Gong

**Affiliations:** Modern Bridge Institute of Structures Technology, Huanghe S&T University, Zhengzhou 450006, Henan, China

**Keywords:** Solid mechanics, Stocky beam theory, Constrained torsion of arbitrary noncircular cross-sectional beams, Nodal-line method, Box beams, Special-shaped columns

## Abstract

The constrained torsion problems of stocky beams, being still an unsurmountable challenge in solid mechanics, will be solved by the innovative method proposed, and the computational results of numerical examples signifying that the beam model proposed is reasonable and the numerical analysis methodology of nodal line method is effective. The creative cruxes are as followings:

• a type of innovative beam model—stocky beam in solid mechanics analysis is defined, which uses the stress state of the beam to distinguish whether a structural member is a beam or not, and the axis of the stocky beam is the locus of the minimum torsional stiffness centre of all its cross sections;
• the warpages of all cross sections of a stocky beam are depicted as a mathematical expression that signifies a family of curved surfaces evolved from Prandtl's membrane shapes;
• the constrained torsion problem of a stocky beam can be transformed into the boundary value problem of a set of ordinary differential equations by using the nodal line method developed.

## Highlights

• Stocky beam theory is developed.
• The constrained torsion problems of stocky beams, being still an unsurmountable challenge in solid mechanics, will be solved by the innovative method proposed.
• The warpages of all cross sections of a stocky beam are expressed as a family of curved surfaces.
• The constrained torsion problem of a stocky beam can be transformed into the boundary value problem of a set of ordinary differential equations.
• The measures for the evaluation of the effectiveness of the innovative method proposed are presented.

## Graphical abstract

**Figure:**
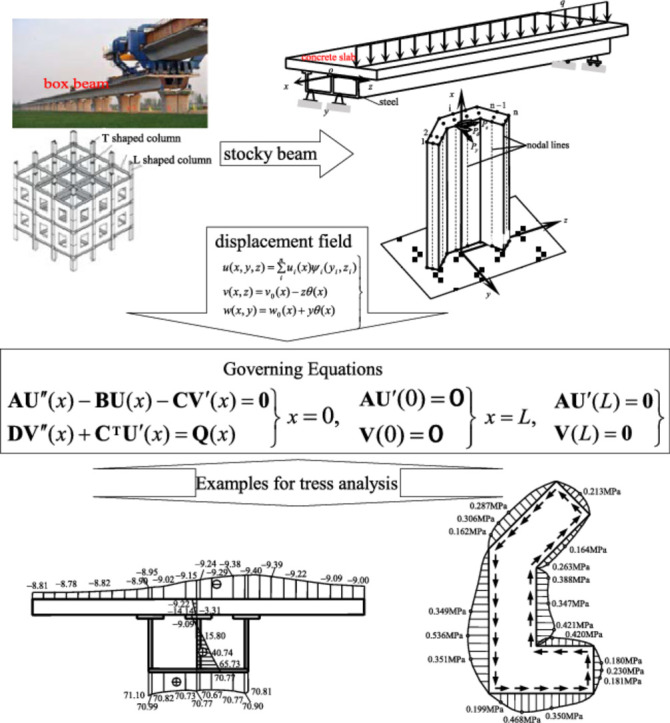

Specifications table

**Table:** 

Subject Area	Engineering
More specific subject area	*Beams*
Method name	*An innovative method for surmounting the constrained torsional problems of stocky beams with arbitrary noncircular cross-sectional shapes and with arbitrary elastic material properties*
Name and reference of original method	H. Cao and Y. Gong*, 2021. The torsional centre position of stocky beams with arbitrary noncircular cross-sectional shapes and with arbitrary elastic material properties. EJMSOL 85 (2021) 104121.http://www.elsevier.com/locate/ejmsol Cheng, X., Chen, H., Gong*, Y., & Yang, Y. B., 2018. Stocky thin- or thick-walled beams: Theory and analysis. Eng. Struct. 59, 55–65.https://doi.org/10.1016/j.engstruct.2017.12.027.
Resource availability	*Yuan S., 1991. ODE conversion techniques and their applications in computational mechanics. Acta Mech Sinica. 7, 283–288.*

**Method details**

## Engineering background of stocky beam

**Fig. 1:**
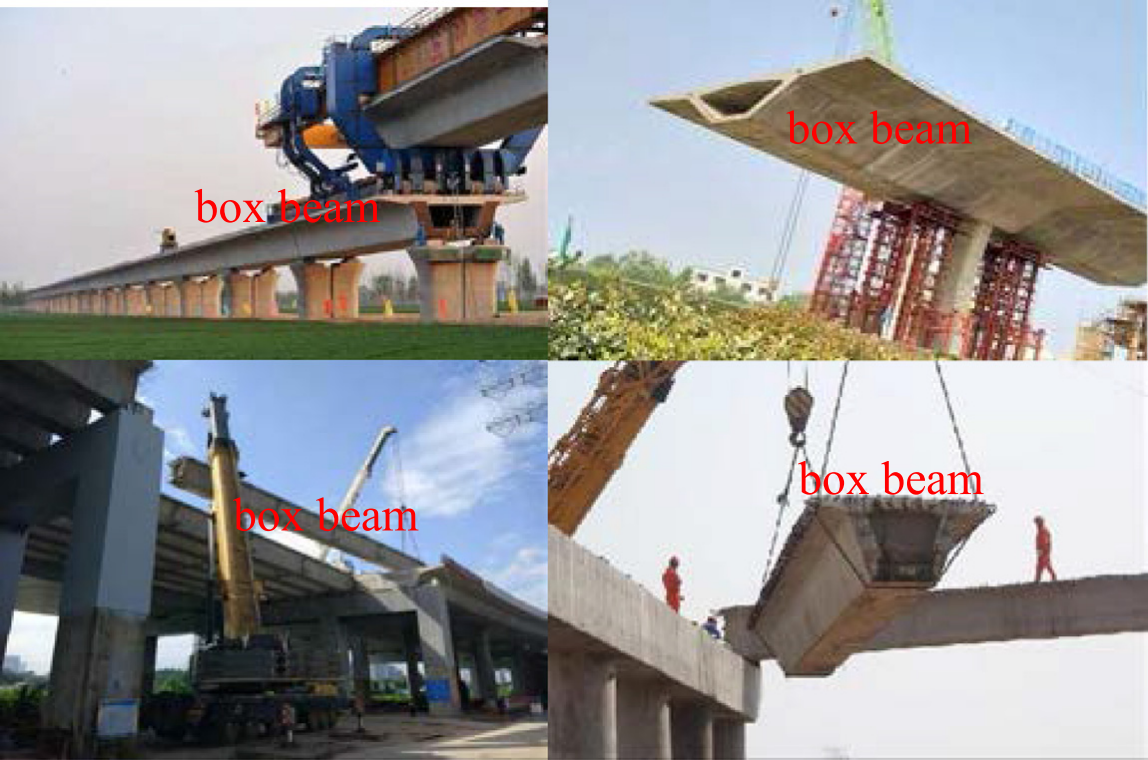
The box beams adopted by high-rise bridges.

**Fig. 2:**
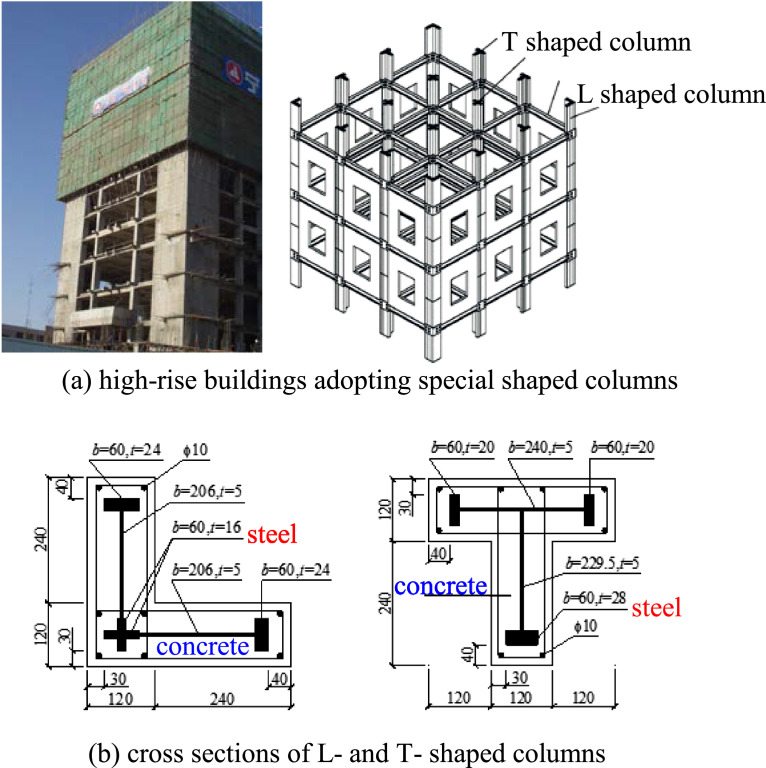
The special shaped columns adopted by high-rise buildings.

The stocky beam involved in this method is the solid-mechanics model for two kinds of structural members in contemporary engineering practice. The first type of the members is the box beams utilized in high-rise highspeed railways or in high-rise highways (Fig. 1). The second type of the members is the concrete-filled steel tubular (or steel reinforced concrete) special-shaped columns adopted in modern high-rise buildings (Fig. 2).

The common characteristics of these two types of structural members in geometry are: (1) the ratio of length to the maximum transverse dimension (width or height) is less than 5; (2) their cross sections are noncircular configurations.

**Fig. 3:**
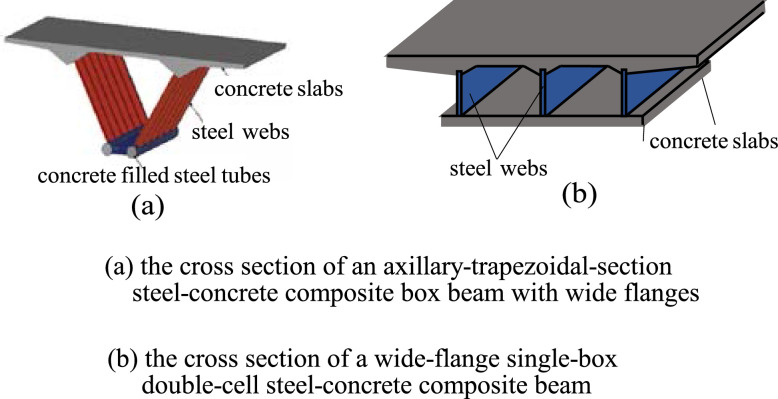
The cross section of box beams.

The cross section of the box beam is neither an open thin-walled cross section nor a closed thin-walled cross section, but a box section with variable wall thickness and wide flange, viz., a combination of a closed box (single- or multi-cell box) with variable wall thickness and two cantilever beams with a variable depth (Fig. 3), and the cross section of a special-shaped column has a thick-walled open configuration (Fig. 2(b).

The common feature of these two types of structural members in material properties is that they are a combination of steel structure and concrete one, that is, they are composite members (Figs. 2,3).

The common working state of these two types of structural members is that they are often staying in the stress state of constrained torsion.

The torsion inducement of the box beam originates from: (1) the nonuniform distribution of vehicle loading in the width direction on the bridge deck; (2) wind loading and seismic loading.

Usually, high-rise highspeed railways or high-rise highways are fundamentally built in mountainous areas, and the distance between the body of the box girder bridge and the bottom of the valley is often hundreds of meters. In this environment, the effects of transverse wind loading and seismic loading on the bridge structure must be considered. And the uncertainty in the acting direction of wind loading and seismic loading will inevitably lead to the torsion of the bridge structure.

**Fig. 4:**
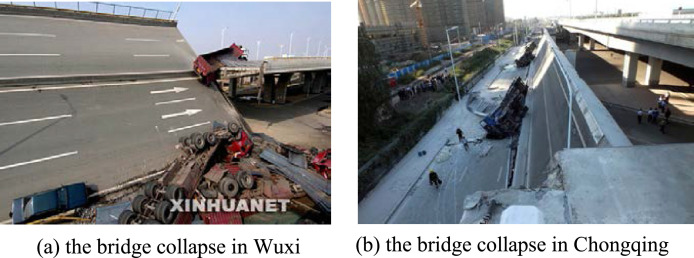
Two instances of collapse of high-rise bridges in China.

In fact, the bridge collapse accidents (see Fig. 4) also verify the correctness of the above two inferences about the inducement of bridge torsion. Although the taking- time and place of the collapse of the two high-rise bridges are different, the inducements of the two collapse accidents are contributed to the torsion of the bridges, from the point of view of solid-mechanics analysis, viz., the torque acting on the whole bridge induced by the external factors exceeds its own anti-torsion ability (the ability to resist overturning).

**Fig. 5:**
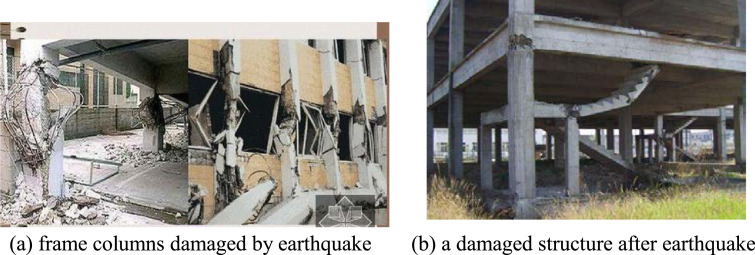
The damage of frame columns stemmed from torsional action of earthquakes.

Like the collapse of bridge structures, the failure form of a high-rise building structure after a shear-wave earthquake is also caused by the torque acting on the whole structural system induced by the external factors beyond its own anti-torsion ability. From the site of the earthquake damage of building structures (see Fig. 5), the most serious damage portion of the whole structural system happens at the four projecting corners of the ground floor, and the failure form of these projecting corner columns is torsional failure.

The above facts show that torsional failure is the most important failure form of high-rise bridge structures and high-rise building structures. However, the traditional structural analysis methods are insurmountable to analyze the mechanics behavior of above-mentioned structural members of modern bridge structures and of super high-rise building under torsion because the cross-sectional shapes and material properties of the members adopted in the structural systems are beyond the application scope of the existing beam theory. Thusly, there is a strong requirement for finding new beam theory and new analysis method for these kinds of structural members.

## Stocky beam theory and nodal line method

**Fig. 6:**
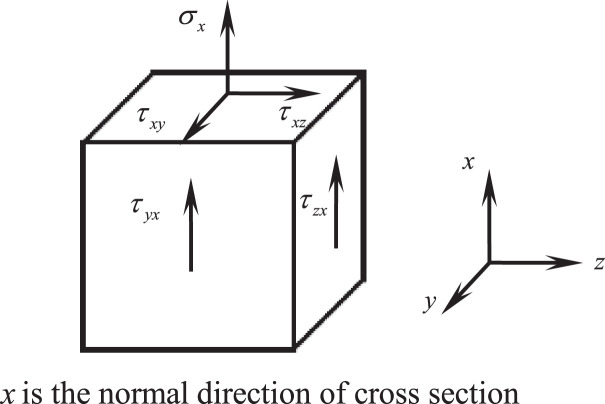
The stress state of an arbitrary point in a beam.
(2) the axis of the beam is the locus of the minimum torsional stiffness centres (MTSC) of all cross sections, not the locus of the geometric centres of all cross sections of the beam. The formula for determining the MTSC is as follows:
  (1) $$\xi_{c}=\frac{\int_{\Omega }G(\xi ,\eta )\xi dA}{\int_{\Omega }G(\xi ,\eta )dA},\eta_{c}=\frac{\int_{\Omega }G(\xi ,\eta )\eta dA}{\int_{\Omega }G(\xi ,\eta )dA}.$$

Stocky beam theory [3] and nodal line method are the appropriate new beam theory and new analysis method that can mend the deficiency of traditional beam theory in analyzing the constraint torsional problem of above-mentioned stocky beams. Compared with the traditional beam theory, the characteristics of the stocky beam theory are as follows:

(1) whether the member is a beam is not distinguished by its aspect ratio, but by the stress state of the member, viz., as long as the stress state of any point in the member only has no more than 3 stresses, i.e., there is only normal stress and shear stress on the cross section while there is no normal stress on the other two pairs of lengthwise sections perpendicular to the cross section of the member (see Fig. 6), the member can be recognized as a beam.

For the meaning of the symbols in the formula and the creation process of the formula, please refer to the literature [2].

**Fig. 7:**
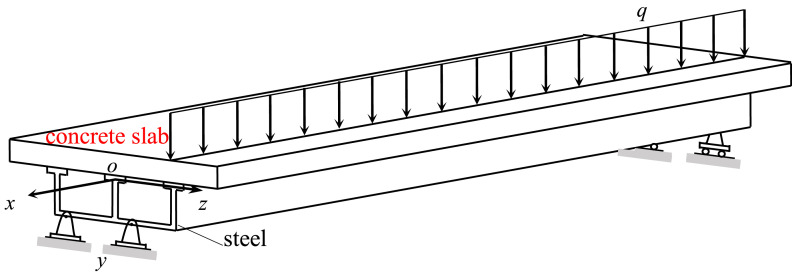
A simply supported composite box beam subjected to a distributed eccentric load.

(3) when establishing the out-of-plane strain law of the cross-section under stress, the plane-section hypothesis utilized by the traditional beam theory is no longer employed, but the mathematical approximation method is adopted to develop the out-of-plane strain law of the cross-section of a beam after deformation. For example, the out-of-plane deformation of the beam shown in Fig. 7 can be expressed as
  (2) $$u\left(x,y,z\right)=\sum_{i}^{n}u_{i}\left(x\right)\psi_{i}\left(y_{i},z_{i}\right).$$

The geometric meaning of this mathematical expression is a family of curved surfaces. $u_{i}\left(x\right)$,i=1,2,⋯,n, represent the elongation or shortening in the *x* direction of the lengthwise lines parallel to the *x* axis (the connections of all MTSC), respectively.

For a specific cross section (say $x=x_{0}$), $u\left(x_{0},y,z\right)=\sum u_{i}\left(x_{0}\right)\psi_{i}\left(y_{i},z_{i}\right)$ expresses one curved surface.

From the point of view of computational mechanics, these *n* lengthwise lines can be recognized as nodal lines performing a semi-discrete treatment to a beam. The unknown functions ($u_{i}\left(x\right)$,i=1,2,⋯,n) representing the longitudinal displacement of these *n* nodal lines are actually the *n* basis functions employed to describe the out-of-plane deformation shapes of all cross sections of the beam. The interpolation functions ($\psi_{i}\left(y_{i},z_{i}\right)$,i=1,2,⋯,n) depicting the displacement between nodal lines are evolved from the membrane shape of Ludwig Prandtl [9], and their unified expression is

(3) $$\psi_{i}\left(y_{i},z_{i}\right)=\left(1-\frac{y_{i}}{w_{i}}\right)\left(1-\frac{z_{i}}{d_{i}}\right),\;i=1,\;2,\;\cdots ,\;n.$$

The meaning of the symbols in the formula is the same as that explained in literature [2]. The so-called Ludwig Prandtl membrane is the analogy membrane used by Ludwig Prandtl in the study of the torsion of thin-walled bars with noncircular cross sections.

(4) On the premise of ignoring the in-plane deformation of the cross section, the crosswise displacements of the beam caused by various factors (shearing, bending, torsion) are expressed as
  (4) $$\begin{array}{c} v\left(x,z\right)=v_{0}\left(x\right)-z\theta \left(x\right)\\ w\left(x,y\right)=w_{0}\left(x\right)+y\theta \left(x\right) \end{array}\},$$

in which, $v_{0}\left(x\right)$and $w_{0}\left(x\right)$ are two unknown basis functions employed to depict the lateral translations of *x* axis in the *y* direction and in the *z* direction, respectively; θ(x) is the unknown basis function used to describe the rigid rotation of the cross sections around the *x* axis.

v(x,z)and w(x,y) separately signify the displacement in the *y* direction and in the *z* direction at any point (*x, y, z*) on the cross section of the beam caused by the coupling action of shearing, bending and torsion. The main reason for the coupling of bending and torsion is that the actual rotational centre (MTSC) of the cross section during torsion is not consistent with the ideal torsional centre (or torsional centre used in traditional beam theory). When the two centres are inconsistent, if the cross section does not rotate about the axis connected by the ideal torsional centres, the beam will produce bending deformation as well as torsional deformation; if the torque acting on the cross section does not rotate about the ideal torsional centre, the beam will produce bending deformation as well as torsion deformation [1,[4], [5], [6].

(5) The concepts of neutral axis, centroid principal axes, moment of inertia of cross section about neutral axis, and etc. used in traditional beam theory and related to plane section hypothesis; and the concepts of sector area and sector pole in Vlasov beam theory will no longer exist in stocky beam theory. The stiffness characteristics related to the deformation of the cross section of a stocky beam are expressed by 4 stiffness matrices: the matrix of tension-compression stiffness, of bending stiffness, of warping stiffness and of shear-torsion coupling stiffness.

## Mathematical models pertinent to the constrained torsion problems of stocky beams

After the description of the displacement mode of the stocky beam under the possible stress state, including the restrained torsion, viz., on the basis of the mathematical expressions (2) and (4), the strain field at any point in the beam can be obtained as follows

(5) $$ɛ=\left[\begin{array}{ccc} \varepsilon_{xx}=u_{,x} & \gamma_{xy}=v_{,x}+u_{,y} & \gamma_{xz}=w_{,x}+u_{,z}\\ \gamma_{yx}=u_{,y}+v_{,x} & \varepsilon_{yy}=v_{,y}=0 & \gamma_{yz}=w_{,y}+v_{,z}=0\\ \gamma_{zx}=u_{,z}+w_{,x} & \gamma_{zy}=v_{,z}+w_{,y}=0 & \varepsilon_{zz}=w_{,z}=0 \end{array}\right],$$

where a comma following a symbol indicates the partial derivative with respect to the coordinate that follows.

Based on following three assumptions, the elastic potential energy of the beam and the work done by the load, the mathematical model of the problem can be deduced according to the energy principle in elasticity.

*Assumptions*

(a) The displacements of any one of beams after deformation are very small vis-a-vis the dimensions of that beam before deformation.
(b) The in-plane rigidity of the cross section is large enough to disregard its distortion.
(c) The delamination of composite beams is neglected.

For example, the ordinary differential equations for the mechanics behavior of a composite box girder (see Fig. 7) subjected to asymmetric vehicle loads are

(6) $$\begin{array}{c} {\mathrm{AU}}^{\prime \prime }\left(x\right)-\mathrm{BU}\left(x\right)-{\mathrm{CV}}^{\prime }\left(x\right)=0\\ {\mathrm{DV}}^{\prime \prime }\left(x\right)+C^{T}U^{\prime }\left(x\right)=Q\left(x\right) \end{array}\},$$

(7) $$x=0,\;\begin{array}{c} {\mathrm{AU}}^{\prime }\left(0\right)=0\\ V(0)=0 \end{array}\},$$

(8) $$x=L,\;\begin{array}{c} {\mathrm{AU}}^{\prime }\left(L\right)=0\\ V(L)=0 \end{array}\}.$$

**Fig. 8:**
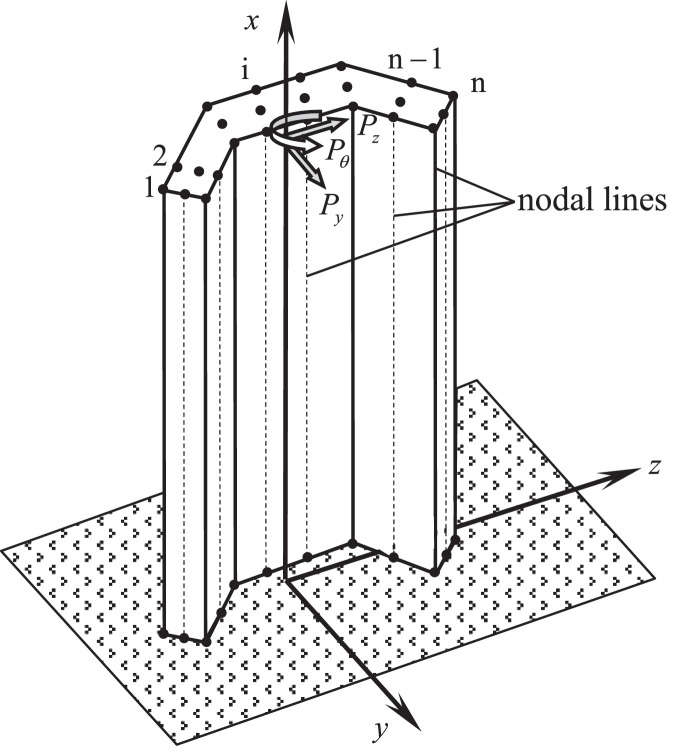
A stocky column with C-shaped cross section subjected to the combined action of transverse forces and torque at its top end.

Similarly, the ordinary differential equations and corresponding boundary conditions for the mechanics behavior of C-section special-shaped reinforced concrete columns with a 3-metres height (see Fig. 8) under the combined action of top transverse forces and a concentrated torque can be expressed as follows

(9) $$\begin{array}{c} {\mathrm{AU}}^{\prime \prime }\left(x\right)-\mathrm{BU}\left(x\right)-{\mathrm{CV}}^{\prime }\left(x\right)=0\\ {\mathrm{DV}}^{\prime \prime }\left(x\right)+C^{T}U^{\prime }\left(x\right)=0 \end{array}\},$$

(10) $$x=0,\;\begin{array}{c} U(0)=0\\ V(0)=0 \end{array}\},$$

(11) $$x=L,\;\begin{array}{c} {\mathrm{AU}}^{\prime }\left(L\right)=0\\ {\mathrm{DV}}^{\prime }\left(L\right)+C^{T}U\left(L\right)-P=0 \end{array}\}.$$

In which,

$$U\left(x\right)=\left\{\begin{array}{c} u_{1}\left(x\right)\\ u_{2}\left(x\right)\\ \vdots \\ u_{n}\left(x\right) \end{array}\right\},\;V\left(x\right)=\left\{\begin{array}{c} v_{0}\left(x\right)\\ w_{0}\left(x\right)\\ \theta (x) \end{array}\right\},\;Q\left(x\right)=\left\{\begin{array}{c} q_{y}\left(x\right)\\ q_{z}\left(x\right)\\ q_{\theta }\left(x\right) \end{array}\right\},\;P=\left\{\begin{array}{c} P_{y}\\ P_{z}\\ P_{\theta } \end{array}\right\}.$$

**A, B, C, D** are the cross-section tension-compression stiffness matrix, anti-bending stiffness matrix, anti-warping stiffness matrix, anti-shear-torsion stiffness matrix, respectively. The form of matrix **A, B, C, D** is the same as that showed in the appendix of literature [2].

## The methodology pertinent to the solution of mathematical model

Evidently, the mathematical models of the former two kinds of problems are boundary value problems of ordinary differential equations (ODE). The numerical solution of boundary value problems of ODEs is introduced in detail in reference [8]. The relationship between the solution accuracy of mathematical model and the correctness of the four stiffness matrices **A, B, C** and **D** will be presented here.

The solution accuracy of the mathematical model is related to the number of nodal lines (or nodes) used in calculating the four stiffness matrices **A, B, C** and **D**. The higher the requirement for the accuracy of the solution, the more the number of nodal lines (or nodes) employed in calculating the four stiffness matrices **A, B, C** and **D**. Generally speaking, if the stress distribution on the cross section of the beam is required, more nodes will be needed; if the overall displacement of the beam is desired, the number of nodes can be less.

The calculation precision of the four stiffness matrices **A, B, C** and **D** directly affects the solution accuracy of the mathematical model, i.e., the solution accuracy of unknown functions ($u_{i}\left(x\right)$,i=1,2,⋯,n) and its derivatives. And the main factor affecting the calculation precision of the four stiffness matrices **A, B, C** and **D** is the selection of interpolation functions $\psi_{i}\left(y_{i},z_{i}\right)$,i=1,2,⋯,n.

The selection of interpolation functions does not have arbitrariness, the selection should not only contemplate thoroughly the out-of-plane deformation shape of the cross section after torsion, but also extract valuable information about the warping configuration of the cross section from previous studies on the torsion problem of noncircular cross-sectional beam. After a large number of computing and checking processes (that is, every computational result must be compared with the existing analytical solutions), the most reasonable interpolation function could be summarized.

My years of research experience signifies that if the interpolation function represented by formula (2) is selected, the numerical solution of the mathematical model will be comparable to the analytical solution (see the verification example 2 in the literature [2]) because formula (2) is a mathematical evolvement to Prandtl's membrane [9] utilized in the study on the maximum shearing stress of beams with noncircular thin-walled cross sections due to torsion.

The computational complexity of the four stiffness matrices **A, B, C** and **D** closely associates with the number of nodal lines or nodes (the intersection of the nodal line and the cross section) employed in formula (2), while the number of nodes is related to the distribution of the material properties, to the geometric shape and to the geometric size of the cross section of the beam. In other words, the more complex the distribution of the material properties on the cross section of the beam (or the more complex the expression of elastic modulus E(y,z) and G(y,z)), the more the number of nodes; the more complex the geometry of the cross section, the more the number of nodes; the larger the size of the cross section, the more the number of nodes. The more the number of nodes, the higher the complexity of the computation of the four stiffness matrices.

The dependence of **A, B, C** and **D** stiffness matrices on the interpolation function determines that their quantitative calculation must be completed by computer programs. Because each interpolation function given in formula (3) is defined only on the small region composed of its adjacent nodes, thusly the calculation of each element in the four stiffness matrices **A, B, C** and **D** must be completed on the small region composed of each node and its adjacent nodes, and then all the elements of the whole stiffness matrix are obtained by the global integration method.

**Fig. 9:**
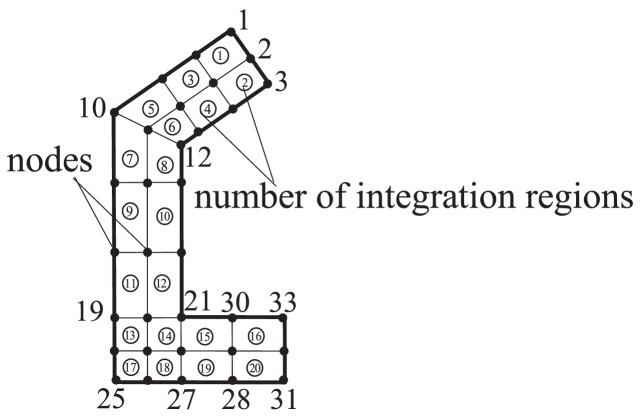
The numbers of nodes and of small integration regions of a C-shaped cross section.

For instance, the calculation of 4 stiffness matrixes **A, B, C** and **D** involved in the bending-torsion coupling problem induced by the action of two transverse loads and a concentrated torque at the free end of a C-shaped column shown in Fig. 8, are assembled by the results integrated on 20 small regions surrounded by 33 nodes shown in Fig. 9.

When compiling computer program for the calculation of the 4 matrices, the main attention should be paid on how to realize the calculation of each element of the 4 stiffness matrices, and then on how to utilize the relationship between the local code number and global code number of the small region to assemble the 4 matrices.

## Concrete steps for analyzing the constrained torsion problem of stocky beams

The concrete steps for analyzing the problem of constrained torsion of stocky beams will be presented as follows.

(1) Determine the location of the minimum torsional stiffness centre (MTSC) by using formula (1).
(2) Establish a global coordinate system whose *x* axis is the locus of all MTSCs.
(3) Select nodal lines parallel to the *x* axis.
(4) Set up a mathematical model for the problem.
(5) Calculate 4 stiffness matrices **A, B, C** and **D** and the load vector, associated with the mathematical model.
(6) Solve the numerical solution of unknown functions and its derivatives in the mathematical model.
(7) Determinate the mechanics behavior of the stocky beam quantitatively according to the calculation results of step (6).

If the mechanics behavior of the beam under the condition of pure constrain torsion is desired, the first target will become to determine the position of the torsional centre of the cross section according to the method introduced in reference [2], and then take the connections of all the torsional centres of the cross sections as the *x*-axis and establish a global coordinate system. Then successively take above steps (3)-(7), the mechanics behavior of the beam under the condition of pure constraint torsion will be obtained.

## Computational results of two numerical examples

Based on above mentioned methodology and analytical steps, constrained torsional problems of stocky beams with arbitrary noncircular cross-sectional shapes and with arbitrary elastic material properties can be surmounted.

**Fig. 10:**
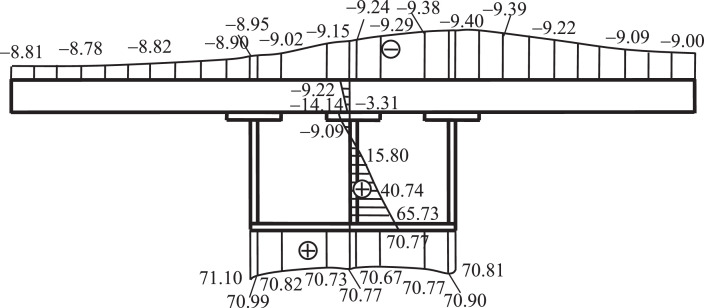
The normal stress distribution of midspan section of the box girder under a uniformly distributed eccentric load (unit: MPa).

**Fig. 11:**
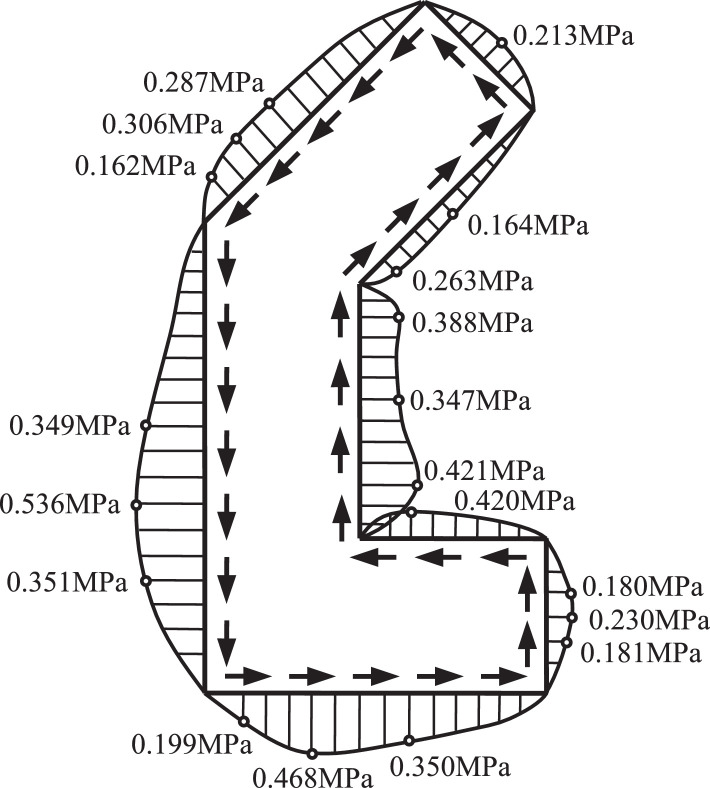
The shearing stress distribution on the cross section of midspan of a reinforce concrete column due to a concentrated torque acting at the top end.

For example, Fig. 10 shows the normal stress distribution on the midspan cross section under a uniformly distributed eccentric load applying to the deck of the composite box girder shown in Fig. 7. And Fig. 11 indicates the shearing stress distribution on the midspan cross section under the action of a concentrated torque acting at the top end of the column shown in Fig. 8, with a generalized C shape.

The correctness of these computational results can be validated by the measures presented in the following section.

## Evaluation of the effectiveness of the method

As delineated in section 4, the solution accuracy of the mathematical model is related to not only the number of nodal lines used in calculating the four stiffness matrices A, B, C and D, but also the calculation precision of the four stiffness matrices. Therefore, the calculation accuracy of these four stiffness matrices is very important to the final computational results of the mathematical model of the problem analyzed. In order to ensure that the calculation accuracy of the four stiffness matrices does not affect the accuracy of the final computational results of the mathematical model of the problem analyzed, the following approaches will discuss how to evaluate the calculation accuracy of the four stiffness matrices.

(1) compare numerical solution with analytical one

The analytical solution mentioned here is the analytical solution of the bending problems of Bernoulli-Euler beams because they always exist [7].

For the stocky beams studied in this paper, although their aspect ratios do not meet the application conditions of Bernoulli-Euler beams, the stiffness matrices A, B, C and D, which reflect the stiffness characteristics of the cross section, are only related to the size, to the shape and to the elastic modulus of materials of the cross section, but not to the length of the beam. Therefore, in order to evaluate whether the stiffness matrices A, B, C and D can reflect the real stiffness characteristics of the cross section of a stocky beam, the length of the stocky beam can be lengthened to make it to have the length of a new Bernoulli-Euler beam, and the numerical solution of the deflection of the new Bernoulli-Euler beam under a given transverse load can be computed by the method proposed in the paper, and then the numerical solution of the deflection can be compared with its analytical one calculated by the associated formula of the new Bernoulli-Euler beam. If the two solutions are completely consistent, or very close to each other, then the computational results of stiffness matrices A, B, C and D are reasonable and effective, and then the four matrices can be employed to analyze the mechanics problems related to the stocky beam.

When making the above comparison, if encountering that a stocky beam whose cross section has a nonuniform material property, a stiffness equivalence can be achieved to the beam to make it to be of a uniform material property, and then the comparison can be completed.

(2) evaluate the correctness of the solution according to the objective facts

For the constrained torsion problems of some new types of components encountered in contemporary civil engineering, there might be no way to compare the numerical results computed by using the method proposed in the paper with the numerical ones of other methods or the experimental ones because the problems themselves have gone beyond the ability scope of the existing beam theory or of the experimental approaches. In this situation, the rationality and validity of the computational results of this kind of problems can be evaluated by the objective facts that should be obeyed by this kind of problems.

For example, for the constrained torsion problem of unequal-limb L-shaped concrete filled steel tubular (or steel reinforced concrete) columns, when analyzing the constrained torsion behavior of this kind of members under the action of a pure torque, the position of the torsional centre of their cross section must be determined first. However, the cross section of this kind of members is an open thick-walled section without a symmetrical axis, which is also composed of more than two kinds of materials, and the conventional method and the existing method improved on the grounds of conventional beam theory is insurmountable to determine its torsional centre position. It is also intractable to procure the position of the torsional centre of the cross section by experimental methods.

In this condition, the torsional centre of the cross section of an equal-limb L-shaped concrete filled steel tubular (or steel reinforced concrete) column can be determined first. The determination of the torsional centre position of the cross section of the equal-limb L-shaped column must first utilize the computer program to calculate the four stiffness matrices A, B, C and D of the cross section, and then apply the method proposed to determine the coordinates of the torsional centre position of the cross section. If the coordinates determined are reasonable, then they must be located on the 45-degree symmetry line of the cross section, otherwise, they will be unreasonable, because an objective fact must be followed by the equal-limb L-shaped column is that the 45-degree line of its cross section is the symmetrical line of the size, of the shape and of the material properties of its cross section. The torsional centre as one of the inherent characteristics of the cross section must also be located on the 45-degree symmetry line. This is the objective fact that a reasonable calculation must not violate.

When the computer program for calculating the four stiffness matrices A, B, C and D of the cross section of the equal-limb L-shaped column has passed the above evaluation, the torsional centre position of an unequal-limb L-shaped concrete filled steel tubular (or steel reinforced concrete) column can be calculated by the computer program evaluated. After the numerical solution of the coordinates of the torsional centre position of the cross section of the unequal-limb L-shaped column is procured, the rationality and validity of the numerical solution can also be evaluated macroscopically according to the size, to the shape and to the material properties of the cross section, and the numerical solution whether accord with the objective facts or not can be judged thusly.

For example, the torsional centre of an unequal L-shaped concrete filled steel tubular (or steel reinforced concrete) column should approach the side of the long limb part of the cross section because the stiffness of the long limb part of the cross section is larger than that of the short limb part, being an objective fact of the inherent hallmarks of the cross section of this kind of member.

The above two approaches to evaluate the correctness of the computational results have been applied in the literature [2] through examples, and interested readers can find more details about the evaluation method in this reference for in-depth understanding.

## Conclusion remarks

The problem of constrained torsion of composite members with thick-walled noncircular cross sections in contemporary civil engineering is still an unsurmountable challenge in solid mechanics. This paper presents the methodology to surmount this challenge. The main points are summarized as follows.

(1) An innovative solid mechanics model, stocky beam, is presented in this paper. the stocky beam theory can be applied to two kinds of structural members in contemporary civil engineering structures. The first kind of structural members is the box beams adopted in the high-rise highspeed railway or highway, and the second kind of structural members is a variety of special-shaped columns adopted in contemporary tall building structures.
(2) It is introduced how to use the nodal line method to express the out-of-plane warping shape of all the cross sections of a stocky beam after constrained torsion, viz., the mathematical method how to express a family of curved surfaces is developed.
(3) The analysis steps for solving the constrained torsion problem of stocky beams are discussed, and the computational results about stress analysis of two torsion-bending coupling problems of two examples are also presented so as to illustrate the power of the innovative method.
(4) The measures for the evaluation of the effectiveness of the innovative method proposed are presented.

## Declaration of Competing Interest

The authors declare that they have no known competing financial interests or personal relationships that could have appeared to influence the work reported in this paper.
